# Low-Spin States From Decay Studies in the Mass 80 Region

**DOI:** 10.6028/jres.105.006

**Published:** 2000-02-01

**Authors:** J. Döring, A. Aprahamian, M. Wiescher

**Affiliations:** Department of Physics, University of Notre Dame, Notre Dame, Indiana 46556

**Keywords:** low-spin states, neutron deficient nuclei, prolate deformation

## Abstract

Neutron-deficient nuclei in the mass 80 region are known to exhibit strongly deformed ground states deduced mainly from yrast-state properties measured in-beam via heavy-ion fusion-evaporation reactions. Vibrational excitations and non-yrast states as well as their interplay with the observed rotational collectivity have been less studied to date within this mass region. Thus, several *β*-decay experiments have been performed to populate low-spin states in the neutron-deficient ^80,84^Y and ^80,84^Sr nuclei. An overview of excited 0^+^ states in Sr and Kr nuclei is given and conclusions about shape evolution at low-spins are presented. In general, the non-yrast states in even-even Sr nuclei show mainly vibration-like collectivity which evolves to rotational behavior with increasing spin and decreasing neutron number.

## 1. Introduction

There is now extensive experimental evidence for large prolate deformation in the neutron-deficient Rb, Sr, and Y nuclei. For the even-even Sr isotopes, the evidence is based on experimental quadrupole moments extracted from level lifetimes [1,2] and excitation energies of the first excited yrast states [3]. In all these neutron-deficient nuclei, the underlying cause of the prolate deformation has been attributed to the population of strongly polarizing orbitals originating from the *d*_5/2_ and/or intruder *g*_9/2_ subshells and large gaps in the single-particle level energies.

The evolution of shapes of mass 80 nuclei from near-spherical to γ-soft and to well-deformed shapes as function of particle number and angular momentum has been investigated using different theoretical approaches [4–6]. In some cases, shape coexistence interpretations have been invoked to describe irregularities of the moments of inertia of some neutron-deficient even-even Se, Kr, and Sr nuclei at low spins [7]. For the even-even Sr isotopes the situation is quite complicated. Large prolate deformations as observed for ^76,78^Sr are in agreement with most of the recent calculations while the nucleus ^80^Sr is predicted to be spherical in the ground state with *β*_2_ = 0.053 [6]. The ground-state deformation of *β*_2_ ≈ 0.4 as deduced from in-beam γ-ray experiments [1,2] is in contrast to recent results from fast beam laser spectroscopy [8] where the deduced mean charge radii indicate somewhat less deformed shapes for ^78,80^Sr. The neutron-deficient even-even Sr isotopes exhibit yrast level sequences (or moments of inertia) at low spins which show large deviations from the behavior expected for a rigid rotor, possibly indicating shape fluctuations. Thus, the issue of the rigidity of the shapes and the occurrence of co-existing configurations are not yet resolved and have not been thoroughly addressed as many of the key states of interest are of low spins and of non-yrast nature, i.e., they are not well populated in the heavy-ion fusion reactions usually used for the in-beam studies.

Properties of nuclei along the *N* = *Z* line are also of interest for the astrophysically relevant rapid proton capture (rp) process [9] which is thought to be one of the dominant energy sources in cataclysmic binaries like novae and x-ray bursts. The rp process is characterized by a sequence of fast proton capture reactions and subsequent *β* decay. Usually, the *β* decay is slow compared to the fast proton capture reactions. Waiting points can develop where the proton capture is compensated by inverse photo-disintegration or where single proton capture is inhibited at the proton-drip line. The lifetimes of these waiting-point nuclei are determined by the *β* decay of the ground state or thermally excited states. Thus lifetimes of ground states and/or *β*-decaying isomeric states in the vicinity of the proton-drip line are important input parameters for calculations of nuclear synthesis, luminosity, and time scale [10]. Nucleosynthesis at the extreme temperature and density conditions associated with such events may well proceed beyond the doubly-magic ^56^Ni [11].

Only few alternative probes are available for investigating non-yrast states in nuclei far from stability. The most useful is the careful investigation of the *β* decay from a higher-*Z* parent nucleus. The parent spins are usually low so a large number of non-yrast states is expected to be populated when the decay energy is large. For a successful *β*-decay experiment sufficient production of the parent nuclei is needed. Far from stability, this is experimentally difficult as production cross section are small and the nuclei are short-lived.

## 2. Low-Lying Isomers in the Odd-Odd ^80,84^Y Isotopes

### 2.1 New Isomer in ^80^Y

A new *β*-decay experiment has been performed to study the low-spin structure of the *N* = *Z* + 2 nucleus ^80^Y. The ^80^Y source has been produced via the fusion-evaporation reaction ^24^Mg(^58^Ni,pn) reaction at 190 MeV. The use of inverse kinematics provided a strongly forward-peaked recoil spectrum best suitable for an efficient collection and subsequent separation by the Argonne fragment mass analyzer [12]. The *A* = 80 mass separated recoils were implanted on a plastic tape and transported to a *β*- and γ-ray counter station consisting of three Ge detectors and a low-energy photon spectrometer. Each γ-ray detector had a thin plastic scintillator in front for the detection of *β* rays. The recoils were implanted within a deposition time of 20 s and their radioactive decay was subsequently measured for 20 s. Several cycles were also performed with 60 s deposition time and 60 s counting time. More experimental details have been reported in Ref. [13].

**Fig. 1:**
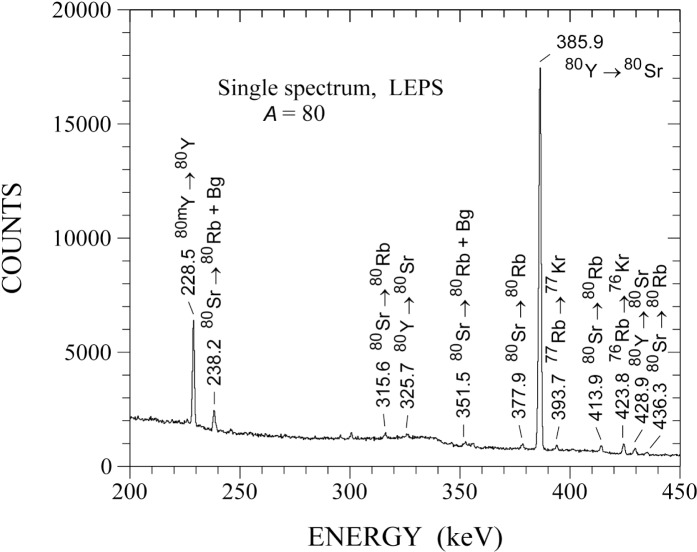
Single γ-ray spectrum recorded with a low-energy photon spectrometer. The mass 80 recoils were mass separated by the Argonne fragment mass analyzer and transported to the counter station by a moving tape system. The figure has been taken from Ref. [13].

**Fig. 2:**
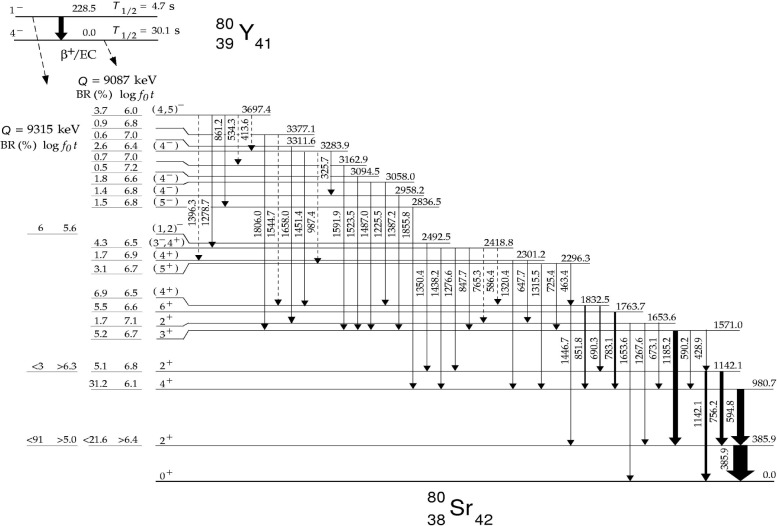
Level scheme of ^80^Sr deduced from the *β* decay of ^80^Y. The figure has been taken from Ref. [14].

**Fig. 3:**
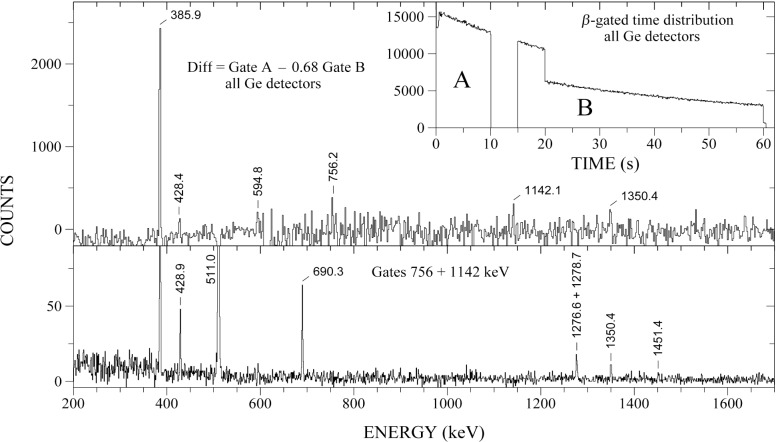
Difference spectrum (top panel) of *β*-gated events from all Ge detectors to illustrate the decay of the 1^−^ isomer in ^80^Y. The gating conditions are shown in the inset. To obtain the best possible statistics, the events from both 20 s and 60 s cycles have been added up causing the visible step at the time of 20 s. The 756 keV and 1142 keV background-corrected sum coincidence spectrum is shown in the bottom panel. The figure has been taken from Ref. [14].

A single γ-ray spectrum recorded with the low-energy photon spectrometer and representative for the decay of the short-lived mass 80 recoils is displayed in Fig. 1. The strongest γ-ray peak has been identified as the 2^+^ → 0^+^ transition in ^80^Sr. Further, a new γ-ray transition at 228.5 keV has been found [13] which is the second strongest line in the spectrum. This transition depopulates a new isomer in ^80^Y with a half-life of 4.7(3) s [13]. Spin and parity of the isomer has been determined to be 1^−^. Thus, the isomer decays by a M3 transition to the 4^−^ ground state. The extracted M3 transition strength is 0.78(5) Weisskopf units. Most interestingly, the isomer undergoes *β* decay as well to low-lying states in ^80^Sr [14], as can be seen in the decay scheme of the isomer given in Fig. 2, upper left-hand side. This conclusion has been drawn from two experimental facts: (i) The time distribution of the 2^+^ → 0^+^ 385.9 keV transition in ^80^Sr does not show the expected delayed feeding by the 228.5 keV isomeric transition (as the 4^+^ → 2^+^ 594.8 keV transition does), i.e., the time distribution can be fitted well with a single exponential decay curve. This indicates that the delayed component is canceled out. (ii) The difference spectrum between early and late time correlated events exhibits a strong 385.9 keV transition. This spectrum is shown in Fig. 3. The spectrum has been generated by subtracting the time-γ events of the 15 s to 60 s time range (late events) from the time-γ events of the 0 s to 10 s range (early events). Further, events in the time range 10 to 15 s have been excluded (see inset of Fig. 3). For normalization, we assumed that the intensity of the 783.1 keV line depopulating the 6^+^ state at 1763.7 keV in ^80^Sr cancels out leading to a factor of 0.68. As a result a small intensity amount of the 594.8 keV line remains in the difference spectrum. This may indicate that the 1^−^ isomeric *β* decay is highly fragmented. The situation is similar to the 1^−^ ground-state *β* decay of ^76^Rb [15]. The difference spectrum indicates, in addition to the strong 385.9 keV transition, a weak 1350.4 keV line. The same 1350.4 keV transition can be seen in the sum coincidence spectrum of the 756 and 1142 keV gates providing evidence for a level at 2492.5 keV. This level seems to be populated in the isomeric decay only and has probably a low spin.

The *β*-decay branch has been estimated to be about 19(2) %. This result has important consequences for calculations of the rp-process nucleosynthesis of ^80^Kr since the longer lived ground state of ^80^Y (*T*_1/2_ = 30.1(5) s [13]) is partly bypassed by the isomeric *β* decay, and a shorter effective half-life of ^80^Y is obtained which leads to a reduction of the calculated overproduction of ^80^Kr [10].

Total Routhian surface calculations [4] have shown that the odd-odd nucleus ^80^Y exhibits a strongly deformed prolate shape with a quadrupole deformation of *β*_2_ = 0.37 for the ground state. The prolate minimum persists up to high rotational frequencies. Thus, the deformed shape inspired the application of two-quasiparticle-plus-rotor calculations to investigate the wave functions of the low-lying states in terms of Nilsson orbitals. We found that the low-spin structure can be well explained if a proton-neutron residual interaction is employed. In this case the ordering of the states and the energy splitting between the 4^−^ ground state and the 1^−^ isomer can be well reproduced. The wave functions contain mainly the proton [422]5/2^+^ and the neutron [301]3/2^−^ Nilsson orbitals. These orbitals are coupled parallel and antiparallel in the 4^−^ ground state and in the 1^−^ isomer of 80Y, respectively. The model calculations demonstrate that the deformed picture accounts very well for the observed properties of the low-lying states in ^80^Y.

### 2.2 Low-Spin States in ^84^Y

Early evidence was presented that the odd-odd nucleus ^84^Y has very likely an 1^+^ ground state and a higher-lying (5^−^) isomer at an energy of about 500 keV [16,17]. This structure was deduced from early decay studies and the excitation energy of the isomer was an estimate only. Also, a few γ rays had been previously assigned to the ^84^Zr decay [18], however, not placed into a level scheme. Therefore, three new decay experiments have been carried out: (i) via the irradiation of a ^58^Ni target with ^28^Si ions at 97 MeV using a modified NORDBALL setup [19], (ii) via the irradiation of a ^58^Ni target with 99 MeV ^28^Si ions and (iii) via the irradiation of a ^58^Ni target with 135 MeV ^32^S ions [20]. The latter two experiments were performed at Florida State University. In the first two experiments the chosen target-projectile combinations ensured that the even-even nucleus ^84^Zr was produced in-beam, without any in-beam population of states in ^84^Y and ^84^Sr. In this way all states seen in these two latter nuclei were populated via the *β*-decay chain ^84^Zr → ^84^Y → ^84^Sr only. The experiments at Florida State University were carried out with 5 Ge detectors and a low-energy photon spectrometer to detect the γ rays.

**Fig. 4:**
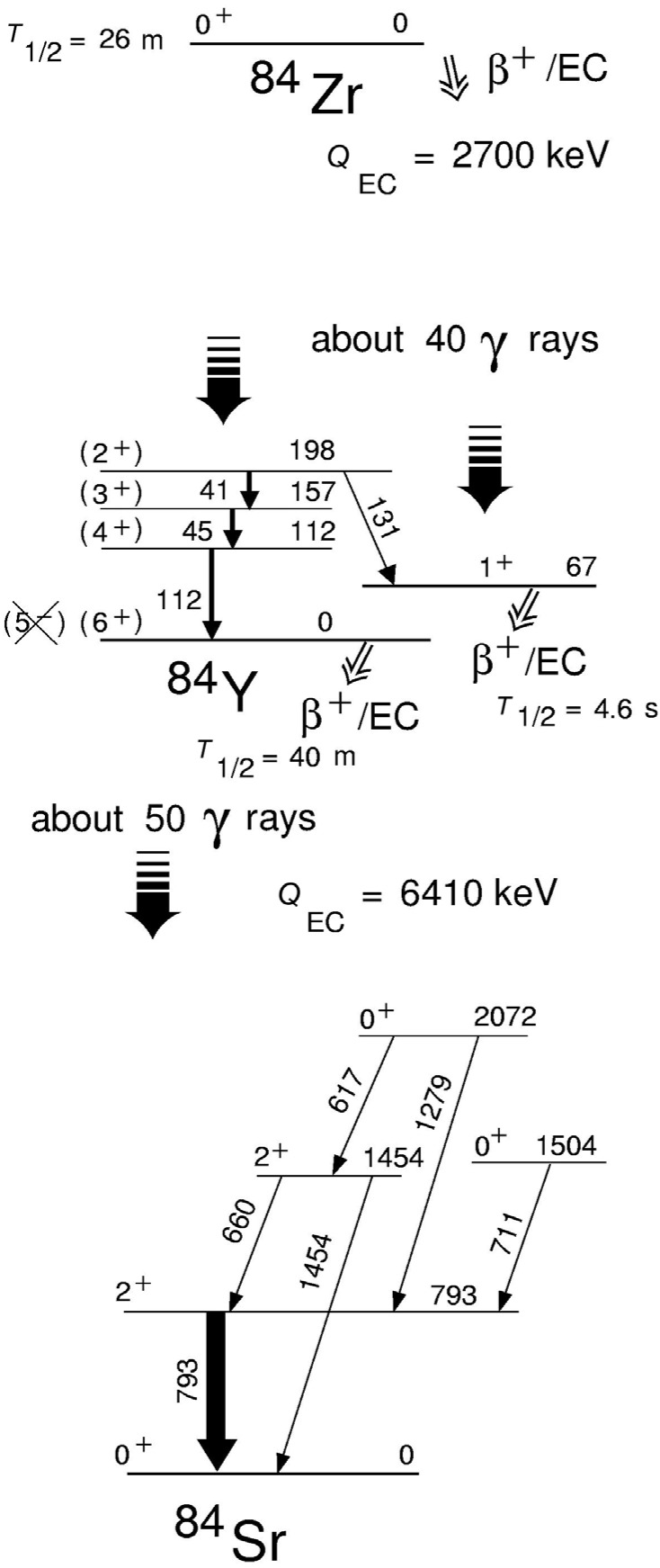
Selected low-lying states in odd-odd ^84^Y and even-even ^84^Sr observed in *β* decay via the chain ^84^Zr → ^84^Y → ^84^Sr using five Ge detectors and a low-energy photon spectrometer. The experimental results have been taken from Refs. [19,20].

It has been found that the 1^+^ isomer in ^84^Y has an excitation energy of 67 keV and undergoes *β* decay only. No low-energy 67 keV γ transition to the ground state in ^84^Y has been seen in the singles spectrum measured with the low-energy photon spectrometer. A partial decay scheme is shown in Fig. 4 where emphasis has been placed on the low-spin structure in ^84^Y and the population of the 0^+^ states in ^84^Sr by the *β* decay of the 1^+^ isomer. Further, states up to (7^+^) in the γ-vibrational band of ^84^Sr have been identified giving evidence for a possible spin and parity assignment of 6^+^ to the ground state of ^84^Y, in contrast to the previous assignment of (5^−^) [17].

The new decay data revealed many new γ rays in ^84^Y and ^84^Sr and hence many new levels have been identified in both nuclei. For example, the previously reported excited 0^+^ states at 1505 and 2075 keV in ^84^Sr as identified via a (p,t) reaction [21] have been observed via γ-ray spectroscopy at 1504 and 2072 keV, respectively, for the first time. These states depopulate via 711 and 1279 keV transitions to the first excited 2^+^ state at 793 keV in ^84^Sr. An intense 793 keV peak has been seen only in the coincidence gates at 711 and 1279 keV indicating a very low multiplicity. Thus the origin is very likely a low-spin state in ^84^Y, i.e., the *β* decay of the 1^+^ isomer. The number of coincidence events of the 1279 keV line gated by the 793 keV transition in the 10 different detector-pair matrices of experiment (iii) was good enough to deduced angular correlation coefficients [22]. They provide evidence for a 0^+^ → 2^+^ → 0^+^ decay sequence.

## 3. Low-Lying States in Even-Even Neutron-Deficient Sr and Kr Isotopes

### 3.1 Excited 0^+^ States in Sr Isotopes

The evolution of the nuclear shape from spherical to deformed in the even-even Sr isotopes is well known when moving away from the neutron shell closure at *N* = 50. These findings are based mainly on yrast level properties investigated via heavy-ion fusion-evaporation reactions. The study of non-yrast low-lying states may provide additional evidence to support these claims, or may indicate a more complex nuclear structure at low spins. The careful study of the *β* decay of odd-odd Y study of the *β* decay of odd-odd Y nuclei seems to be the best method for populating non-yrast levels in neutron-deficient even-even Sr isotopes. Thus, the experiment described before for the investigation of an isomer in ^80^Y has been analyzed for the ^80^Y → ^80^Sr *β* decay as well. The high selectivity of the Argonne fragment mass analyzer and the use of a multi-detector setup provided clean data. The known ^80^Sr level scheme could be extended by 14 new levels [14], see Fig. 2. Spin and parity assignments are given based on the observed feeding and depopulation pattern, deduced log *ft* values, and on a comparison with the decay of the ^78^Rb 4^−^ isomer to low-lying states in ^78^Kr [23].

**Fig. 5:**
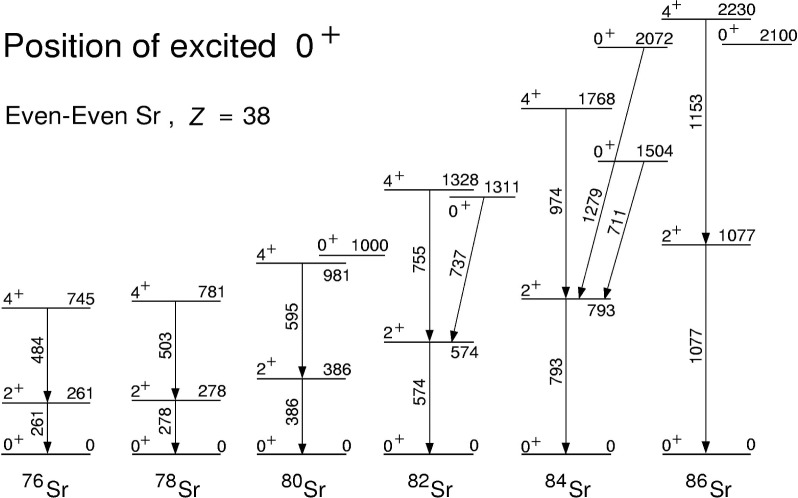
Excited 0^+^ states and the lowest yrast excitations are displayed for even-even neutron-deficient Sr isotopes. The experimental results on the 0^+^ states have been taken from: ^80^Sr, Ref. [29]; ^82^Sr, Ref. [17]; ^84^Sr, Refs. [20,21]; ^86^Sr, Ref. [21].

Most of the known excited 0^+^ states in mass 80 nuclei have been identified via radioactive decay studies or particle-transfer reactions. The experimental detection is sometimes difficult since a $0_{2}^{+}\to 0^{+}$ E0 transition can be verified only via a conversion electron measurement. Using γ-ray spectroscopy, usually the $0_{2}^{+}\to 2_{1}^{+}$ E2 transition is detected. In general, the E0 matrix elements depend sensitively on the nuclear charge distribution and thus on the nuclear deformation [24]. Hence, the identification of these excited 0^+^ states in a chain of isotopes allows to study the evolution of the nuclear shape at low spins. The latest results for the even-even Sr isotopes (*Z* = 38) are displayed in Fig. 5. The previously reported 0^+^ states in ^84^Sr, detected via particle-transfer reactions and confirmed by present γ-ray spectroscopy, are included. With decreasing neutron number, the position of the excited 0^+^ states decreases as well and a multiplet-like grouping of the levels is obtained.

### 3.2 Excited 0^+^ States in Kr Isotopes and N = 38 Isotones

**Fig. 6:**
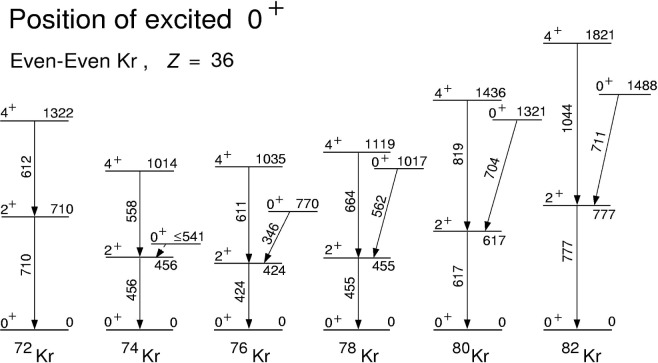
Excited 0^+^ states and the lowest yrast excitations are displayed for even-even neutron-deficient Kr isotopes. The experimental results on the 0^+^ states have been taken from: ^74^Kr, Ref. [25]; ^76,78,80,82^Kr, Ref. [17].

The systematics of the excited 0^+^ states in the neutron-deficient even-even Kr isotopes is plotted in Fig. 6. The recently discovered low-lying $0_{2}^{+}$ state in ^74^ Kr, at most 85 keV above the first excited 2^+^ state at 456 keV [25], refines the previously suggested shape coexistence picture [26]. This picture of a deformed-spherical shape coexistence was invoked to explain the irregularities in the energy spacings (or moments of inertias) of the lowest yrast excitations in the even-even ^74,76^Kr nuclei. Now an oblate shape is suggested for the excited 0^+^ state in ^74^Kr, in contrast to the prolate deformed ground-state band. The half-life reported for the $0_{2}^{+}$ in ^74^Kr is the partial time for the E0 transition. The low-energy γ-ray decay has not been found yet.

**Fig. 7:**
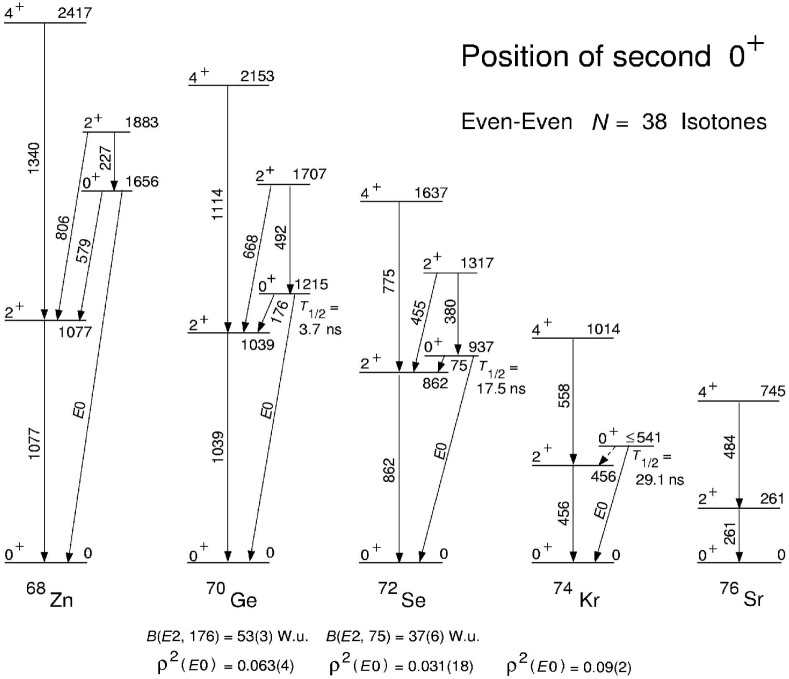
Excited 0^+^ states in some neutron-deficient *N* = 38 isotones. The experimental E2 and E0 transition strengths are given. The data have been taken from: ^70^Ge, Ref. [27]; ^72^Se, Ref. [28]; ^74^Kr, Ref. [25].

It should be pointed out that the second 0^+^ state in ^74^Kr fits quite well into the *N* = 38 systematics as can be seen in Fig. 7. In most of these isotones, an excited 0^+^ state has been found which decays by a low-energy γ ray to the first 2^+^ state. The deduced $0_{2}^{+}\to 2^{+}$ E2 transition strengths are in the order of 45 Weisskopf units indicating substantial collectivity. The reported E0 matrix elements are also given in the figure.

### 3.3 Vibration-Like Multiplets in Even-Even Sr Nuclei

**Fig. 8:**
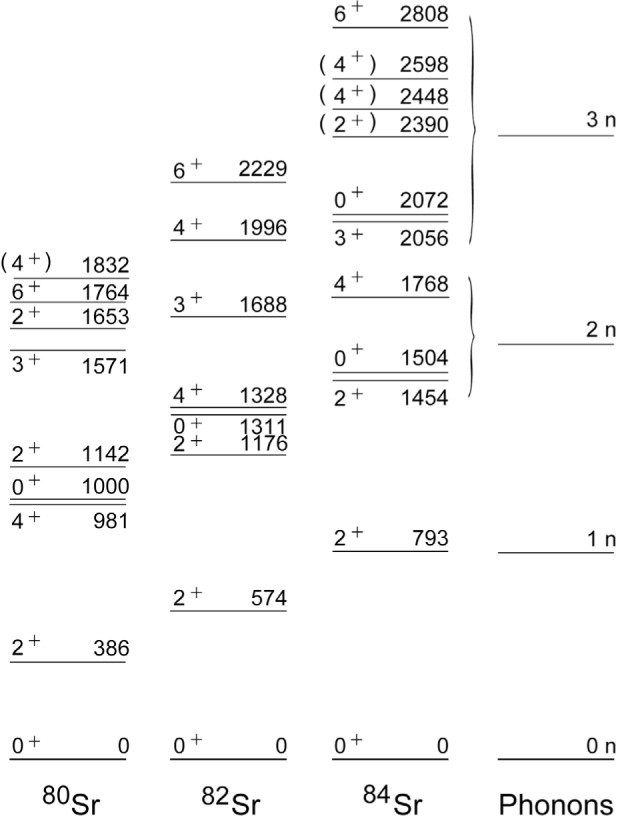
Low-lying levels in the even-even ^80,82,84^Sr isotopes. The level energies indicate the vibration-like multiplet structure. For ^84^Sr, the multiple one-phonon energies are given on the right-hand side.

As can be seen in Fig. 8, the new level scheme of ^80^Sr deduced from our *β*-decay study is clustered into states typical of one-, two-, and three-phonon multiplets of an anharmonic vibrational nucleus. In this approach the lowest 2^+^ state at 385.9 keV can be interpreted as an one-phonon vibrational state. States corresponding to the two-phonon triplet may be the observed states with spins $2_{2}^{+}$ and $4_{1}^{+}$ at energies of 1142.1 and 980.7 keV, respectively. From theoretical considerations there should also be a 0^+^ state to complete the two-phonon triplet. A 0^+^ level at 1.0 MeV was observed in a ^78^Kr(^3^He,n)^80^Sr reaction study [29] but this level has not been seen in our decay data set. Based on a phenomenological parametrization of the effective interaction between phonons [30,31] and using experimental values for the interaction parameters as deduced from members of the observed three-phonon multiplet, a range of 820 keV to 880 keV can be estimated for the excitation energy of the two-phonon 0^+^ state. For three phonons, the expected multiplet of levels consists of $0_{3}^{+},2_{3}^{+},3_{1}^{+},4_{2}^{+}$, and $6_{1}^{+}$. There are observed states with $2_{3}^{+},3_{1}^{+},4_{2}^{+}$, and $6_{1}^{+}$ at 1653.6 keV, 1571.0 keV, 1832.5 keV, and 1763.7 keV, respectively, which might be identified with these excitations. The expected $0_{3}^{+}$ level has not been seen. Similar to the estimate of the excitation energy of the $0_{3}^{+}$ state, an energy range of 1890 keV to 2270 keV can be deduced for the third 0^+^ state based on the anharmonicity of the $2_{2}^{+}$ state.

The observed vibrations in ^80^Sr are clearly anharmonic since the (2*I* + 1) weighted energy centroids of the known members of the multiplets are at 1036 keV and 1726 keV for *n* = 2 and 3, respectively, i.e., the higher orders (with *n* = 2, 3) are not strictly a multiple of the one-phonon energy of 386 keV. The deviations from the expected energies for a harmonic vibrator can be attributed to various anharmonic effects. One such anharmonicity may arise from a finite quadrupole deformationor angular momentum dependence of the nuclear shape. Much less anharmonicity is needed to understand the low-lying states in ^84^Sr, as can be seen on the right-hand side of Fig. 8. In particular, the observed 0^+^ states fit very well into this interpretation and complete the multiplets. The energy centroids of the *n* = 2, 3 multiplets are almost a multiple of the 793 keV (*n* = 1) energy. Thus, an almost harmonic vibration-like nature in ^84^Sr is deduced.

## 4. Summary and Conclusions

Modern *β*-decay experiments employing multi-Ge detector and scintillator arrays combined with in-flight mass separation of recoils produced via nuclear reactions provide a very sensitive tool for the investigation of low-spin states in nuclei far off the line of stability. This has been demonstrated by the recent results obtained for the highly-fragmented radioactive decay of ^80^Y → ^80^Sr. In general, the new decay data suggest that the low-lying structures of ^80,84^Sr show many vibration-like features in a potential with modest deformation including candidates for two- and three-phonon multiplets. This vibration-like nature seems to evolve to a more rotational behavior with increasing angular momentum and decreasing neutron number.
